# Prevalence and Potential Predictors of Frailty among Community-Dwelling Older Persons in Northern Thailand: A Cross-Sectional Study

**DOI:** 10.3390/ijerph17114077

**Published:** 2020-06-08

**Authors:** Payom Thinuan, Penprapa Siviroj, Peerasak Lerttrakarnnon, Thaworn Lorga

**Affiliations:** 1 Department of Community Medicine, Faculty of Medicine, Chiang Mai University, Chiang Mai 50200, Thailand; payom_th@cmu.ac.th; 2 Department of Family Medicine, Faculty of Medicine, Chiang Mai University, Chiang Mai 50200, Thailand; peerasak.lerttrakarn@cmu.ac.th; 3 School of Nursing, Mae Fah Luang University, Chiang Rai 57100, Thailand; thaworn.lorga@gmail.com

**Keywords:** frailty, prevalence, predictors, community-dwelling, older persons

## Abstract

This study aimed to determine the prevalence and associated factors of frailty among Thai older persons. A cross-sectional study was conducted with a representative sample of 1806 older persons aged 60 years or older. Frailty was assessed by Fried’s frailty phenotypes, which consists of five criteria, namely, unintended weight loss, exhaustion, slow walking, weak handgrip and decreased physical activity. Older people who met 3 in 5, 1–2 in 5, and none of the criteria were considered frail, pre-frail and non-frail respectively. The prevalence was calculated and multinomial logistic regression was performed. Prevalence rates of frailty, pre-frailty and non-frailty were 13.9% (95% CI 9.9 to 18.8), 50.9% (95% CI 47.5 to 54.1) and 35.1% (95% CI 31.5 to 39.9), respectively. Increasing age, lower education, having no spouse, poorer health perception, increasing number of comorbidities, osteoarthritis and smaller mid-arm circumference increased the risk of frailty (*p* < 0.001). The prevalence of geriatric frailty syndrome in this study was much higher than that of developed countries but was lower than that of less developed countries. Factors associated with frailty reflect common characteristics of disadvantaged older persons in Thailand.

## 1. Introduction

Frailty is a commonly recognized geriatric syndrome in clinical practice and one of the challenges for healthcare professionals who care for older people. Frailty is an important health issue as it increases the risk of adverse outcomes, increases health care costs, reduces functional reserve, impairs multisystem functions, and increases hospitalization and death [1,2]. The frailer older people become, the more care they need [3]. Most studies have examined risk factors of frailty in community-dwelling older persons. The prevalence rates of frailty among community-dwelling elderly reported in a systematic review varied from 4% to 59.1% with an overall prevalence of 10.7% [4]. The differences in prevalence rates were due to the use of different screening tools [5]. Studies that measured only physical frailty reported a prevalence of 9.9%, whereas those measuring broad phenotypes of frailty, such as physical functions, cognitive functions, health-related factors, and psychological factors reported a prevalence of 13.6%. Geographical, cultural, and socio-demographic differences also contributed to variations in frailty. With regard to the screening tools, Fried’s frailty phenotypes is a commonly used tool as it addresses physical characteristics of frailty, and is an appropriate assessment tool for frailty in the elderly [6,7,8]. In addition, it renders clinical use and practicality.

Data about the prevalence of frailty among community-dwelling older persons in Thailand is quite limited. The prevalence rates of geriatric frailty in Thai communities have recently been reported in two studies. Again, geographical, socio-demographic and measurement methods explained the difference in prevalence rates in these two Thai studies. The first study, which was a nationwide follow up of 9208 people aged 60 years or over between 2009–2016 and employed the Thai Frailty Index, reported a rate of 22.1% [9]. The index was based on broad frailty phenotypes, namely, medical comorbidity, functional status, and physical and emotional health. The second study reported a frailty prevalence of 15.0% among Thai older persons in two northern provinces of Thailand [10]. The strength of this study was in its use of Fried’s phenotype as a measure of frailty. However, the study sample was limited only to people aged 65–74 years old. The reported prevalence thus may not be generalized to the wider Thai older population, which is generally defined as people aged 60 years and older. Including the sample of older people from all age categories (i.e., young-old, old-old, and oldest old) would have reflected the actual prevalence and thus have more relevant policy and practice implications.

A number of factors have been found to be associated with geriatric frailty. In more developed countries, these factors include age, black race, female gender, cardiovascular disease, number of comorbidities, functional incapacity, poor self-rated health, depressive symptoms, body mass index, smoking, low schooling level, low income, poor cognitive function and alcohol consumption [2,11,12,13,14]. In less developed countries, these factors include age, female gender, lower education, longest-held occupation, lower socioeconomic status, low physical activity, comorbidities, functional status and nutritional status [15,16,17,18]. In summary, factors associated with geriatric frailty fall into the following domains: socio-demographic, physical, psychological, functional, and lifestyle. The previous Thai study did not report factors associated with frailty as defined by Fried’s phenotype.

This study attempted to narrow gaps in knowledge about geriatric frailty in Thailand. It aimed to determine the prevalence of frailty among community-dwelling older persons across all age categories using a clinically applicable tool, namely, Fried’s phenotype. Furthermore, it aimed to examine an association between risk factors (socio-demographic, health, and anthropometric characteristics) and geriatric frailty, and also determine the relevance between these factors and any frailty phenotypes in the Thai community. The identified prevalence will help health policy makers gauge the extent to which health and social service systems need to respond to geriatric frailty amidst rapid population aging. Identification of associated factors will improve understanding of mechanisms related to geriatric frailty in Thai populations. This will eventually lead to the identification of preventive measures to delay geriatric frailty and prevent adverse outcomes resulting from frailty.

## 2. Materials and Methods

### 2.1. Design and Study Population

A cross-sectional study was conducted in Lampang, a province with the second-highest aging index in northern Thailand. We calculated the sample size using n4Studies application to estimate the finite population proportion using the standard formula [19,20]. We used the prevalence of frailty in a previous study of Thailand of 17.5% to estimate the expected proportion (p) of frailty in our study [21]. A total of 1806 individuals were required to measure the prevalence with an absolute precision of 5% and error (d) of 0.02. The inclusion criteria were residence in the sampled villages for least 6 months and agreement to participate in the study. Older persons were excluded if they were totally blind and deaf, bedridden, had disability in both hands (unable to grip), had severe illnesses (such joint inflammation, chest pain, blood pressure more than 150/90 mmHg, severe headache, dizziness), had neurological disease (such as stroke and Parkinson’s disease), or had dementia (Mental State Examination—Thai version—MSET10 less than 10) [22].

Twenty-four villages in three districts were selected to represent urban (eight villages), semi-urban (eight villages), and rural (eight villages) communities. Lists of older people were obtained from primary care unit records. Stratified random sampling was used to select the participants. In case of the unavailability of the participant at the time of data collection, the participant living in the nearest house was selected. A total of 1806 older persons were recruited into the study.

### 2.2. Data Collection and Measurement

We conducted face-to-face assessments at the participant’s home from September 2017 to December 2018. The questionnaire was used to collect data on socio-demographic variables (age, gender, education level, and marital status), self-health rate, comorbidities, self-reported medical diagnoses (such as hypertension, diabetes mellitus, heart disease, chronic lung disease, knee osteoarthritis, poor vision and poor hearing), number of medications used and body mass index; BMI.

Handgrip strength was measured using a handheld dynamometer (Takei TKK5001^®^). Height, mid-arm circumference (MAC) and calf circumference (CC) were assessed using standard tape (Tajima brand, PIT-20BL model) and weight using a calibrated weighting scale (Shaper Disney^®^). CC measurement was obtained over the unclothed area at the largest diameter on the left leg. The tape was pulled snug around the calf line and recorded to the nearest 0.1 cm [23]. All measurements were administrated by 10 field investigators who were trained and the measurements were standardized by the principal investigator.

Frailty was assessed based on Fried’s phenotype [24] which includes five criteria, as follows:

(a) Unintended weight loss: This criterion determined unintended weight loss of at least 4.5 kg over the past 12 months. This was measured by using the following calculation:
  K = (weight self-reported in previous year - current measured weight])/(weight self-reported in previous year).
  If K ≥ 0.05 and the subject does not report that he/she was trying to lose weight (i.e., unintentional weight loss of at least 5% of previous year’s body weight), this indicated a positive finding for frailty in terms of weight loss.
(b) Exhaustion: This was determined through self-report using Fried’s method of assessment. The participant was first asked to self-assess whether she/he felt exhausted. If yes, she/he would be asked to rate the severity of the exhaustion. Ratings of 2 to 4 suggested a positive assessment.
(c) Slow walking: This was determined through Fried’s method of assessment. The participant was asked to walk a 15-foot path. The measurement was considered in conjunction with height and sex. Positive assessment was suggested if she/he met one of the following criteria:
  1. Height ≤ 173 cm and walk time ≥ 7 s for men
  2. Height > 173 cm and walk time ≥ 6 s for men
  3. Height ≤ 159 cm and walk time ≥ 7 s for women
  4. Height > 159 cm and walk time ≥ 6 s for women
(d) Weak handgrip: Fried’s method of assessing grip strength weakness was determined by considering grip strength in relation to BMI and sex. This was considered inappropriate in Thai older people as Thai and western elderly populations differ in their BMI and hand size. We therefore did not use Fried’s method of handgrip assessment in this study. Instead, we used the handgrip criterion recommended by the Consensus Report of the Asian Working Group for Sarcopenia. The participant was asked to use the non-dominant hand to perform handgrip. Positive assessment was suggested if the handgrip was less than 26 kg for men and less than 16 kg for women [25].
(e) Decreased physical activity: Fried’s method of assessing physical activity was based on the short version of the Minnesota leisure time activity questionnaire. This was deemed inappropriate for Thai older populations as a number of activities described in the questionnaire were different from those performed by Thai people in their day-to-day living. We therefore used the SHARE-FI questionnaire, which was based on self-reports and has been used in previous studies on frailty [26,27,28]. The SHARE-FI focused on low and moderate physical activity and was found to correlate well with other measures of physical activity. The participant was asked to rate the frequency of their involvement in low or moderate physical activity. Positive assessment was suggested if she/he performed these physical activities three times or fewer in a month [26].

Frailty was then determined by considering all the criteria. Participants who met 3–5 criteria were frail; those with 1–2 were pre-frail. Participants who did not meet any criteria were non-frail [24].

### 2.3. Statistical Analysis

Data were analyzed using SPSS version 22 (SPSS Inc., Chicago, IL, USA) for all of the statistical analyses and a *p*-value of 0.05 was used. Baseline characteristics were compared between frailty, pre-frailty and non-frailty groups using Chi-square or Fisher’s exact tests. The Kolmogorov–Smirnov test was used to verify the normal distribution and the data were normally distributed. First, we used univariate logistic regression to examine all potential factors associated with frailty. The variables with *p*-value < 0.05 on univariate analysis were then selected for multinomial regression analysis with backward elimination. In order to assess the relative contributions of these associated factors to frailty, we conducted the analyses based on three different models. Model 1 was based on personal socio-demographic factors (i.e., age, education level, and marital status). Model 2 included the factors in Model 1 plus health characteristic factors (i.e., self-health rate, number of comorbidities, polypharmacy, and self-reported medical diagnoses such as hypertension, knee osteoarthritis, poor vision and poor hearing). Model 3 combined all factors in models 1 and 2 plus anthropological factors (i.e., BMI and mid-arm and calf circumferences). Results were presented as odds ratios and 95% confidence intervals.

### 2.4. Ethical Considerations 

The study was approved by the Research Ethics Committee of the Faculty of Medicine, Chiang Mai University, Thailand (No. 236/2015), in accordance with the Declaration of Helsinki. All the participants provided written informed consent to participate in this study.

## 3. Results

Study participants had an age range of 60–93 years and a mean age of 70.74 (SD ± 7.46) years. The majority were female (Female = 1274, Male = 532) and had finished primary school (*n* = 1326). Overall, 959 (53.1%) were married and 721 were either widowed, divorced, or single. The mean number of comorbidities was 1.38 (SD ± 1.23). Most of them reported moderate health (*n* = 937), used 1–3 medications (*n* = 979), were overweight or had a BMI ≥ 23.0 kg/m2 (*n* = 1779), had a calf circumference of more than 31.01 cm (*n* = 1261), and had a midarm circumference of more than 22.01 cm (*n* = 1620).

### 3.1. Prevalence of Frailty of Older Persons

A total of 1806 older persons were enrolled. The prevalence of frailty was 13.9% (*n* = 252). The prevalence rates for pre-frailty and non-frailty were 50.9% (*n* = 920) and 35.1% (*n* = 634) respectively. The prevalence of frailty was higher in females compared with males. The proportions of pre-frail and non-frail female and male older persons were similar. Rates of positive assessment by individual criteria were similar among females and males, except for low physical activity, which was doubled in males (Table 1).

### 3.2. Risk Factors According to Frailty Status 

Age, education level, marital status, comorbidities, hypertension, knee osteoarthritis, poor vision, poor hearing, self-health rate, number of medications used, BMI and CC and MAC were found to be associated with frailty and pre-frailty (*p* < 0.05) (Table 2).

### 3.3. Predictors Associated with Frailty among Older Persons

This study presented the results of multinomial logistic regression to identify the risk factors associated with frailty status. In a socio-demographic model (Model 1), age (OR = 1.112, 95% CI 1.092 to 1.132), low education including no school (OR = 3.647, 95%CI 2.165 to 6.146) and primary school, (OR = 1.992, 95%CI 1.508 to 2.630) and having no spouse (OR = 1.440, 95%CI 1.153 to 1.799) were associated with pre-frailty. With respect to frailty, age (OR = 1.199, 95% CI 1.170 to 1.229), low education (OR = 3.368, 95%CI 1.889 to 7.205; OR = 1.556, 95%CI 1.018 to 2.413 respectively) and having no spouse (OR = 1.644, 95% CI 1.184 to 2.283) were associated with frailty (Table 3).

In a health-related model (Model 2), age (OR = 1.112, 95% CI 1.092 to 1.132), no school education (OR = 3.540, 95% CI 2.079 to 6.027) and primary school (OR = 2.011, 95% CI 1.512 to 2.675), having no spouse (OR = 1.428, 95% CI1.139 to 1.1791), self-health rate (poor (OR=1.917, 95% CI 1.199 to 3.064 and) and moderate health (OR = 1.324, 95% CI 1.048 to 1.673), number of comorbidities (three or more illnesses) (OR = 0.678, 95% CI 0.465 to 0.455), knee osteoarthritis (OR = 1.691, 95% CI 1.096 to 2.610), poor vision (OR = 1.953, 95% CI 1.215 to 3.138) and number of medications used (four or more medications) (OR = 1.909, 95% CI 1.136 to 3.210) were associated with pre-frailty. With respect to frailty, age (OR = 1.204, 95% CI 1.172 to 1.237), education (no school (OR = 4.002, 95% CI 1.994 to 8.003) and primary (OR = 1.774, 95% CI 1.130 to 2.784)), having no spouse (OR = 1.590, 95% CI 1.133 to 2.231), self-health rate (poor (OR = 5.923, 95% CI 3.307 to 10.607) and moderate health (OR = 1.592, 95% CI 1.098 to 2.307)), number of comorbidities (1–2 illnesses) (OR = 2.234, 95% CI 1.012 to 4.973), knee osteoarthritis (OR = 1.827, 95% CI 1.041 to 3.207) and poor hearing (OR = 2.094, 95% CI 1.050 to 4.179) were associated with frailty.

In a more holistic model (Model 3), age (OR = 1.105, 95% CI 1.085 to 1.126), education including no school (OR = 3.396, 95% CI 1.982 to 5.819) and primary (OR = 1.886, 95% CI 1.413 to 2.518), having no spouse (OR = 1.374, 95% CI 1.092 to 1.728), self-health rate (poor (OR = 1.969, 95% CI 1.229 to 3.156 and moderate health OR = 1.332, 95% CI 1.052 to 1.687), comorbidity (1–2 illnesses) (OR = 0.676, 95% CI 0.461 to 0.990), knee osteoarthritis (OR = 1.790, 95% CI 1.154 to 2.777), poor vision (OR = 1.979, 95% CI 1.226 to 3.196), number of medications used (1–3 medications) (OR = 1.998, 95% CI 1.183 to 3.374), and MAC (OR = 0.917, 95% CI 0.885 to 0.951) were associated with pre-frailty. 

With regard to frailty, age (OR = 1.195, 95% CI 1.165 to 1.227), education (no school and primary) (OR = 3.767, 95% CI 1.866 to 7.607 and OR = 1.685, 95% CI 1.069 to 2.657 respectively), having no spouse (OR = 1.514, 95% CI 1.074 to 2.133), self-health rate poor (OR = 5.948, 95% CI 3.309 to 10.692 and) and moderate health (OR = 1.585, 95% CI 1.092 to 2.301), comorbidity (three or more illnesses), (OR = 2.364, 95% CI 1.059 to 5.277) and knee osteoarthritis (OR = 1.959, 95% CI 1.112 to 3.451) were associated with frailty.

Across the three models, age, education, and having no spouse persisted as factors associated with pre-frailty and frailty. Among Models 2 and 3, self-health rate and knee osteoarthritis persisted as factors associated with pre-frailty and frailty.

## 4. Discussion

This study determined the prevalence of geriatric frailty and its associated factors among community-dwelling older persons aged 60 years and older in a northern Thai province. The prevalence rates of frailty and pre-frailty were 13.9% (95% CI 9.9 to 18.8) and 50.9% (95% CI 47.5 to 54.1), respectively. The prevalence rates reported in this study are comparable to those reported in some Asian countries using Fried’s phenotype amidst differences in methods of individual parameter assessment and age stratification of the samples. All of these studies were conducted with community-dwelling older persons. The prevalence rates of frailty in more developed countries were lower than the prevalence rates found in this Thai study. The prevalences in Singapore, China, Japan, Malaysia and Taiwan were 5.7%, 7.0%, 9.3%, 9.4%, and 11.3% respectively [2,3,12,14,29].

The prevalence in less developed countries such as Sri Lanka, Vietnam, Indonesia and India were 15.2%, 21.7%, 25.2% and 28.0%, respectively [15,16,17,18], which were higher than the present study. The present study also identified a very similar prevalence rate to previously reported in two provinces of northern Thailand, which was a 15.0% frailty prevalence [10]. In addition, a previous study reported that the frailty prevalence in community-dwelling older persons in all parts of Thailand was 22.1%, which was higher than in our study [9]. Interestingly, the prevalence rates of frailty among these Asian countries reflect the level of socioeconomic development of the nations. The prevalence rates seem to be lower in more developed nations; whereas the rates are higher in less developed settings.

The prevalence rates of pre-frailty in this study and the abovementioned Asian countries follow a pattern similar to that of frailty. Pre-frailty prevalence was 2.2%, 43.9%, 48.5%, 51.2%, 57.9%, 61.6%, and 65.6% in Singapore, Japan, Sri Lanka, China, Malaysia, Indonesia, and Vietnam, respectively [3,12,14,15,16,17,29].

Age, low education and having no spouse were associated with pre-frailty and frailty. The results in this study are consistent with those of previous studies. Increasing age increases the likelihood of developing frailty [13]. Advancing age is accompanied with physical and functional decline [30]. These declines inevitably result in eating problems, activity intolerance, loss of muscle mass, impaired gait and balance and physical inactivity [30,31,32].

The latter two socio-demographic variables, low education and having no spouse, reflect common characteristics of the old-old and oldest old Thai populations. Low education may hamper access to information related to self-care [15], thus impeding appropriate actions in preventing and managing frailty such as nutritional management and physical activity engagement.

Having no spouse may reflect the lack of caregivers or social support among these older participants. Lack of caregiving support often leads to poor nutritional status and physical activity disengagement [33]. This in turn leads to physical frailty [34]. Across the three models, age, education, and having no spouse persisted as factors associated with pre-frailty and frailty. Focusing on health issues, perceived poor or moderate health, having 1–2 comorbidities, knee osteoarthritis, and poor hearing were associated with frailty. These findings were consistent with previous studies reported in international literature [35]. Perceived health in older persons is often associated with the ability to eat and perform daily activities [36,37] in qualitative studies. A number of quantitative studies have also reported a positive association between perceived health and appetite, physical agility, and physical mobility [38,39]. Comorbidity has been consistently found to be associated with nutritional status, physical fitness, and functional ability across different cultures [40,41,42]. Knee osteoarthritis, which impairs physical mobility, has been identified as a risk factor for frailty [42,43,44]. Among Models 2 and 3, which deal with modifiable factors, self-health rate and knee osteoarthritis persisted as factors associated with pre-frailty and frailty. Hearing impairment is associated with fear of falling [45], which, in turn, leads to activity restriction [46]. Eating problems, impaired physical mobility, physical fitness, functional ability and activity restriction to a certain extent contribute to frailty.

Polypharmacy has been found to be associated with frailty in other studies [47]. In this study, the number of medications used was not associated with frailty. However, number of medications used (1–3 medications) was found to be associated with pre-frailty, whereas taking four or more medications was not associated with pre-frailty [48]. When the illness is not well controlled and becomes more severe, the number of medications used is prescribed to achieve optimal control. Older persons with severe illnesses often experience functional disability such as difficulty walking and exhaustion thus making them home-bound. Severely ill older persons who were on multiple medications, therefore, did not participate in this study due to our exclusion criteria. We therefore opine that frailty in this older Thai sample might reflect the influence of normal physiological ageing processes and disuse syndrome more than pathological processes.

Unlike other studies, this study did not find an association between anthropometric factors (i.e., BMI, MAC and CC) and frailty in any model [49]. We originally intended to use BMI, MAC and CC to gauge the nutritional status of the participants as they were used in previous research in Taiwan [50]. These three parameters are included in the Mini-Nutritional Assessment (MNA) tool. It appears to us that these parameters alone may not be adequate when used to assess nutrition in older persons in this study. It is also of note that the WHO did not recommend the use of such parameters alone in assessing nutrition in older persons as they are less reliable [23]. In addition, this may also be due to the use of different cut-off points in our study and the Taiwanese study.

Like any study, this study does have limitations. First, our methods of assessment of some of the Fried’s criteria (i.e., handgrip and physical activity) differed from those of previous studies. These differences make it difficult to ascertain the comparability of results across studies. Second, we needed to rely on self-reported data on past body weight and this made assessment of unintended weight loss less accurate. This was inevitable as we had no access to proper and reliable databases or records. To maximize the accuracy of self-reported BW, we asked family members to validate the reported data, mostly by recalling BWs taken during visits to health facilities or at home by health volunteers as very few families had a scale at home. It is of note that these scales used by individual health facilities and families differ in specifications and reliability. We attempted to consult senior citizen club records to validate the data. Unfortunately, only two senior citizen clubs regularly recorded the body weight of their members. The third limitation concerned the study sample itself. As we excluded older persons who were mobility impaired, clinically unfit and cognitively impaired, the prevalence of frailty and pre-frailty may not reflect the actual prevalence among the community-dwelling older population in general. This limitation also applies to the results related to factors associated with frailty.

## 5. Conclusions

The prevalence of frailty among community-dwelling older persons in Lampang Province, Northern Thailand is 13.9%. Frailty prevalence may reflect the level of socioeconomic development where the older persons live. Frailty is a multidimensional condition and is associated with disadvantaged backgrounds such as advancing age, lower education, having no spouse, poorer health, and having mobility-limiting conditions such as knee osteoarthritis.

As the magnitude of geriatric frailty is quite significant, we recommend awareness raising about this condition and its impacts among older persons, family and health professionals. Screening for frailty should be considered in primary care practice, especially in disadvantaged populations. Future research should look at establishing standard assessment tools for frailty in the Thai older population.

## Figures and Tables

**Table 1:** Prevalence and 95% CI of frailty status and each presenting frailty criterion.

	Prevalence (*N*, %) 95% Confidence Interval		
	Overall (*n* = 1806)	Male (*n* = 532)	Female (*n* = 1274)
Frailty status			
Frail (3–5 scores)	252 (13.9) 9.9–18.8	68 (12.8) 10.1–15.9	184 (14.4) 12.6–16.5
Pre-frail (1–2 scores)	920 (50.9) 47.5–54.1	280 (52.6) 48.3–56.9	640 (50.2) 47.5–53.0
Non-frail (0 score)	634 (35.1) 31.5–39.0	184 (34.6) 30.5–38.8	450 (35.3) 32.7–38.0
Frailty criteria			
Weak hand grip	957 (53.0) 49.8–56.2	297 (55.8) 50.0–61.6	660 (51.8) 47.9–55.7
Slow walking	509 (28.2) 24.4–32.4	119 (22.4) 15.5–31.3	390 (30.6) 26.0–35.3
Unintentional weight loss	331 (18.3) 14.4–23.0	92 (17.3) 10.3–26.7	239 (18.8) 14.1–24.4
Decreased physical activity	191 (10.6) 6.5–15.7	82 (15.4) 8.7–25.6	109 (8.6) 3.8–15.1
Exhaustion	165 (9.1) 5.2–14.6	49 (9.2) 3.4–22.2	116 (9.0) 4.2–15.3

**Table 2:** Description of risk factors according to frailty status.

Risk Factors	Overall (*n* = 1806)	Non-Frailty (*n* = 634)	Pre-Frailty (*n* = 920)	Frailty (*n* = 252)	*p*-Value
Age a (years), Mean ± SD, Range	70.74 ± 7.46, 60–93	66.93 ± 5.53, 60–90	71.88 ± 7.26, 60–91	76.11 ± 7.76, 60–92	<0.001 **
60–69	889	447 (50.3)	385 (43.3)	57 (6.4)	
70–79	660	173 (26.2)	382 (57.9)	105 (15.9)	
≥80	257	14 (5.4)	153 (59.5)	90 (35.0)	
Gender ^b^					
Female	1274	450 (35.3)	640 (50.2)	184 (14.4)	0.543
Male	532	184 (34.6)	280 (52.6)	68 (12.8)	
Living area					
Urban	621	220 (35.4)	307 (49.4)	94 (15.1)	0.110
Semi-urban	559	214 (38.3)	269 (48.1)	76 (13.6)	
Rural	626	200 (31.9)	344 (55.0)	82 (13.1)	
Education level ^b^					
No school	161	25 (15.5)	98 (60.9)	38 (23.6)	<0.001 **
Primary school	1326	449 (33.9)	700 (52.8)	177 (13.3)	
Secondary school and higher	319	160 (50.2)	122 (38.2)	37 (11.6)	
Marital status ^b^					
Single	126	46 (36.5)	66 (52.4)	14 (11.1)	<0.001 **
Married	959	405 (42.2)	452 (47.1)	102 (10.6)	
Windowed/divorced/separated	721	183 (25.4)	402 (55.8)	136 (18.9)	
Number of comorbidities ^a^, mean ± SD	1.38 (1.23)	1.17 (1.14)	1.42 (1.26)	1.76 (1.24)	<0.001 **
None	517	209 (40.4)	265 (51.3)	43 (8.3)	
1–2	972	347 (35.7)	482 (49.6)	143 (14.7)	
≥3	317	78 (24.6)	173 (54.6)	66 (20.8)	
Self-reported medical diagnoses					
Hypertension ^b^	816	259 (31.7)	425 (52.1)	132 (16.2)	<0.001 **
Diabetes mellitus ^b^	293	92 (31.4)	155 (52.9)	46 (15.7)	0.380
Heart disease ^b^	77	27 (35.1)	33 (42.9)	17 (22.1)	0.090
Chronic lung disease ^b^	60	18 (2.8)	29 (3.2)	13 (5.2)	0.020 *
Knee osteoarthritis ^b^	180	39 (21.7)	105 (58.3)	36 (20.0)	<0.001 **
Poor vision ^b^	170	33 (19.4)	106 (62.4)	31 (18.2)	<0.001 **
Poor hearing ^b^	112	22 (19.6)	60 (53.6)	30 (26.8)	<0.001 **
Self-health rate ^b^					
Poor	162	34 (21.0)	78 (48.1)	50 (30.9)	<0.001 **
Moderate	937	304 (32.4)	500 (53.4)	133 (14.2)	
Good	707	296 (41.9)	342 (48.4)	69 (9.8)	
Number of medications used ^b^					
None	644	256 (39.8)	321 (49.8)	67 (10.4)	<0.001 **
1–3	979	337 (34.4)	498 (50.9)	144 (14.7)	
≥4	183	41 (22.4)	101 (55.2)	41 (22.4)	
Body mass index ^c^ (kg/m2)					
Underweight (<18.5)	5	1 (20.0)	1 (20.0)	3 (60.0)	<0.001 **
Normal (18.5–22.9)	22	4 (18.2)	11 (50.0)	7 (31.8)	
Overweight (≥23.0)	1779	629 (35.4)	908 (51.0)	242 (27.3)	
Calf circumference ^b^ (cm)					
≤31.00	545	111 (20.4)	334 (61.3)	100 (18.3)	<0.001 **
≥31.01	1261	523(82.5)	586(63.7)	152(60.3)	
Mid arm circumference ^b^ (cm)					
<20.99	57	6 (10.5)	35 (61.4)	16 (28.1)	<0.001 **
21.00–22.00	129	19 (14.7)	76 (58.9)	34 (26.4)	
≥22.01	1620	609 (37.6)	809 (49.9)	202 (12.5)	

^a^ ANOVA; ^b^ Chi-square test; ^c^ Fisher’s Exact test; * *p* < 0.05, ** *p* < 0.001.

**Table 3:** Multinomial regression analysis assessing the association between socio-demographic, health and anthropometric characteristics and frailty.

Characteristics	Model 1		Model 2		Model 3	
	Pre-frailty	Frailty	Pre-Frailty	Frailty	Pre-Frailty	Frailty
	Versus Non-Frail		Versus Non-Frail		Versus Non-Frail	
	OR (95% Confidence Interval)					
Age (years)	1.112 (1.092–1.132) **	1.199 (1.170–1.229) **	1.112 (1.092–1.132) **	1.204 (1.172–1.237) **	1.105 (1.085–1.126) **	1.195 (1.165–1.227) **
Education level						
No school	3.647 (2.165–6.146) **	3.368 (1.889–7.205) **	3.540 (2.079–6.027) **	4.002 (1.994–8.003) **	3.396 (1.982–5.819) **	3.767 (1.866–7.607) **
Primary	1.992 (1.508–2.630) **	1.556 (1.018–2.413) *	2.011 (1.512–2.675) **	1.774 (1.130–2.784) *	1.886 (1.413–2.518) **	1.685 (1.069–2.657) *
Secondary and higher	Reference	Reference	Reference	Reference	Reference	Reference
Marital status						
Married	Reference	Reference	Reference	Reference	Reference	Reference
Windowed/ divorced/ separated/ single	1.440 (1.153–1.799) **	1.644 (1.184–2.283) *	1.428 (1.139–1.791) *	1.590 (1.133–2.231) *	1.374 (1.092–1.728) **	1.514 (1.074-2.133) *
Self-health rate						
Poor health			1.917 (1.199–3.064) *	5.923 (3.307–10.607) **	1.969 (1.229–3.156) **	5.948 (3.309–10.692) **
Moderate health			1.324 (1.048–1.673) *	1.592 (1.098–2.307) *	1.332 (1.052–1.687) *	1.585 (1.092–2.301) *
Good health			Reference	Reference	Reference	Reference
Number of comorbidities						
None			Reference	Reference	Reference	Reference
1–2			0.403 (0.790 0.455)	2.243 (1.012 4.973) *	0.676 (0.461–0.990) *	1.328 (0.739–2.386)
3			0.678 (0.465–0.455) *	1.314 (0.736–2.344)	0.816 (0.467–0.1.424)	2.364 (1.059–5.277) *
Hypertension (Reference = None)			1.141 (0.844–1.544)	0.953 (0.620–1.466)	1.176 (0.867–1.596)	0.996 (0.645–1.537)
Knee osteoarthritis (Reference = None)			1.691 (1.096–2.610) *	1.827 (1.041–3.207) *	1.790 (1.154–2.777) **	1.959 (1.112–3.451) *
Poor vision (Reference = None)			1.953 (1.215–3.138)*	1.198 (0.637–2.225)	1.979 (1.226–3.196) **	1.211 (0.641–2.290)
Poor hearing (Reference = None)			1.480 (0.836–2.618)	2.094 (1.050–4.179) *	1.410 (0.793–2.505)	1.982 (0.989–3.970)
Number of medications used						
None			Reference	Reference	Reference	Reference
1–3			1.157 (0.829–1.613)	1.042 (0.637–1.705)	1.998 (1.183–3.374) *	1.988 (0.976–4.049)
4			1.909 (1.136–3.210) *	1.918 (0.945–3.893)	1.172 (0.838–1.639)	1.043 (0.635–1.711)
Body Mass Index (kg/m^2^)						
Underweight (<18.5)					0.642 (0.018–23.34)	1.588 (0.039–64.876)
Normal (18.5–22.9)					Reference	Reference
Overweight (≥23.0)					2.58 (0.754–8.841)	1.490 (0.359–6.190)
Mid-arm circumference (cm)					0.917 (0.885–0.951) **	0.925 (0.975–1.877)
Calf circumference (cm)					0.999 (0.981–1.027)	0.996 (0.975–1.017)

Model 1 included socio-demographic characteristics; Model 2 included socio-demographic and health characteristics; Model 3 included socio-demographic, health and anthropometric characteristics; * Significant association at 0.05, ** Significant association at 0.001.
